# Evaluation of pharmacodynamic properties and safety of *Cinnamomum zeylanicum* (Ceylon cinnamon) in healthy adults: a phase I clinical trial

**DOI:** 10.1186/s12906-017-2067-7

**Published:** 2017-12-28

**Authors:** Priyanga Ranasinghe, Ranil Jayawardena, Shehani Pigera, Wasundara Sevwandi Wathurapatha, Hasitha Dhananjaya Weeratunga, G. A. Sirimal Premakumara, Prasad Katulanda, Godwin Roger Constantine, Priyadarshani Galappaththy

**Affiliations:** 1 Department of Pharmacology, Faculty of Medicine, University of Colombo, Colombo, Sri Lanka; 2 Department of Physiology, Faculty of Medicine, University of Colombo, Colombo, Sri Lanka; 3 Industrial Technology Institute, Colombo, Sri Lanka; 4 Department of Clinical Medicine, Faculty of Medicine, University of Colombo, Colombo, Sri Lanka

**Keywords:** *Cinnamomum zeylanicum*, Ceylon cinnamon, Efficacy, Pharmacodynamic, Safety, Adverse effects, Sri Lanka, Adults

## Abstract

**Background:**

Cinnamon is considered as a treatment for many ailments in native medicine. Evidence suggests that *Cinnamomum zeylanicum* (CZ) has anti-microbial, anti-parasitic, anti-oxidant, blood glucose lowering properties and beneficial cardiovascular effects. The present study aims to evaluate Pharmacodynamic properties and safety of CZ in healthy adults using a Phase I Clinical Trial.

**Methods:**

This phase I clinical trial was conducted at the Department of Pharmacology, Faculty of Medicine, University of Colombo, Sri Lanka. Thirty healthy adults were recruited for the study, conducted for a period of 3 months, with the dose of CZ (water extract) increased at monthly intervals (85 mg, 250 mg and 500 mg). Data collection was carried out at baseline and during each monthly follow up visit. Anthropometric, clinical and biochemical assessments were done at baseline and during follow up. Adverse effects and drug compliance was also evaluated.

**Results:**

Twenty eight subjects completed the three months follow up. Mean age was 38.8 ± 10.4 years and 50% were males. There were no significant changes in the anthropometric parameters during the three months follow up. Both systolic and diastolic blood pressure reduced significant during the 1st month and this reduction was sustained throughout follow up. Full blood count, renal function tests, liver function tests, fasting blood glucose, HDL-c, VLDL-d and triglycerides remained within the normal range without any significant alteration during the 3 months. A significant reduction in the TC (*p* < 0.05) and LDL-c (*p* < 0.001) was noted at the end of the 3 months follow up period. There were no serious adverse effects (including hypersensitivity) noted. In two participants dyspepsia necessitated the discontinuation of study participation. Drug compliance was between 85 and 95% during the study period.

**Conclusions:**

This is the first phase I clinical trial in health adults evaluating efficacy and safety of CZ. Our results demonstrate no significant side effects and toxicity of CZ, including hepatotoxicity and anti-coagulation properties. CZ demonstrated beneficial anti-hyperlipidaemic and blood pressure lowering effects among healthy adults. Further studies with larger samples and longer durations may be able to elucidate other side effects and better describe the pharmacodynamic properties.

**Trial registration:**

SLCTR/2013/001 (Sri Lanka Clinical Trials Registry: http://www.slctr.lk/trials/106) (Date of Registration: 01/01/2013).

## Background

Cinnamon, the inner bark of a tropical evergreen tree has two main varieties, *Cinnamomum cassia* (*Cinnamomum aromaticum*) and *Cinnamomum zeylanicum* (CZ) [1]*.* In Ayurvedic medicine Cinnamon is considered as a treatment for many ailments, including those of the respiratory, digestive and gynecological systems. *Cinnamomum zeylanicum*, also known as Ceylon cinnamon (the source of its Latin name, zeylanicum) or ‘true cinnamon’ is indigenous to Sri Lanka and Southern parts of India. Sri Lanka (formerly known as Ceylon) produces the largest quantity and the best quality CZ. Preparation of cinnamon involves stripping of the outer bark of the tree and letting the inner bark to dry and curl up into its customary cinnamon quills, it is available in the market either in its whole quill form (Cinnamon sticks) or as ground powder [2]. At present both Cinnamon cassia and CZ are sold as preventative/therapeutic supplements for many ailments including, metabolic syndrome, insulin resistance, type 2 diabetes, hyperlipidaemia and arthritis [2]. It is marketed either as a tablet/capsule or as different items of food fortified with cinnamon, including cinnamon biscuits, tea and sweets.

One important difference between ‘true’ cinnamon and the cassia cinnamon is their coumarin content [3]. The coumarin content in CZ appears to be very small to cause health risks, whereas the coumarin level in *Cinnamomum aromaticum* appears to be much higher and may pose health risks if consumed in higher quantity on a regular basis [3]. The usage of Cassia cinnamon as a regular supplement with meals was not advocated or the daily dosage was restricted in many countries due to the toxic effects of *Cinnamomum aromaticum* on the liver and coagulation [3]. In contrast CZ has shown to contain a lesser content of coumarin [3, 4], and thus it may be possible that CZ could be used in higher doses without toxic effects for longer durations.

A recent systematic review summarized the medicinal properties of CZ [5]. The authors concluded that, the available in-vitro and in-vivo evidence suggests that CZ has anti-microbial, anti-parasitic, anti-oxidant and free radical scavenging properties. In addition the authors also indicated that CZ seems to lower blood glucose, serum cholesterol and blood pressure, suggesting beneficial cardiovascular effects [5]. However they noted the absence of properly conducted randomized controlled human trials, to decide on efficacy and safety of long term CZ use in humans and to determine whether these effects have public health implications. Animal studies on CZ has not demonstrated any significant adverse effects or toxicity on the liver, kidney and the pancreas [6]. However, it is necessary to conduct well planned safety studies in humans, prior to advocating regular use of CZ for any medicinal benefit. This is also required prior to the conduct of medium-long term Randomized Controlled Phase II/III Clinical Trials evaluating the medicinal claims of CZ in humans with disease. The present study aims to evaluate the Pharmacodynamic properties and Safety of CZ in Healthy adults using a Phase I Clinical Trial design.

## Methods

### Study population and sampling

This phase I clinical trial was conducted at the Department of Pharmacology, Faculty of Medicine, University of Colombo, Sri Lanka. The study was approved by the Ethics Review Committee of Faculty of Medicine, University of Colombo, Sri Lanka (EC/12/157) and registered at the Sri Lanka Clinical Trials Registry (SLCTR/2013/001). The clinical trial was conducted in compliance with the Declaration of Helsinki and the Good Clinical Practice (GCP) guidelines. Thirty healthy adults were recruited for the study, based upon inclusion and exclusion criteria (defined below). Sample size for this phase I clinical trial was determined as per the recommendations of the Center for Drug Evaluation and Research at the Food and Drug Administration [7]. The clinical trial was conducted for a period of 3 months, with the dose of CZ increased at monthly intervals. Subjects were initially contacted by open advertisement and informed written consent was obtained from all participants prior to recruitment for the study.

To be included in the study the participants had to be; a) healthy as defined by history, examination (including systolic [SBP] and diastolic blood pressure [DBP]) and baseline investigations (including full blood count [FBC], fasting blood glucose [FBG], serum toat cholesterol [TC], low density lipoprotein cholesterol [LDL-c], high density lipoprotein cholesterol [HDL-c], serum triglycerides [TG], serum creatinine, urine full report [UFR], liver enzymes [AST/ALT], serum bilirubin, coagulation tests [PT/INR] and electrocardiogram [ECG]; b) between age 18–60 years; and c) currently not on any medicines, including nutritional supplement, herbal and/or ayurvedic medicines. Patients with any chronic illness, cirrhosis of the liver and abnormal baseline liver, renal function tests or blood counts were excluded from the study. Female volunteers who were pregnant, lactating or not willing to use an effective form of birth control during the period of study were also excluded.

### *Cinnamomum zeylanicum* Capsules

The treatment drug was a capsule containing CZ as the active ingredient. Ceylon cinnamon quills (Grade H2) required for the extraction process was purchased from a single private estate in Kalupe, in the Southern province of Sri Lanka and its identification was authenticated by an expert (GASP). A voucher specimen was deposited at the Herbarium at the Herbal Technology Section of the Industrial Technology Institute (ITI), Colombo, Sri Lanka (Voucher Specimen Number: HTS-CIN-01 (Ham)). The extraction process was carried out at the Industrial Technology Institute (ITI), Colombo, Sri Lanka. The dried powder of CZ was extracted from stem barks to distilled water using Soxlet apparatus and the resulting hot water extract was freeze-dried to obtain crude water extract from which the capsules were prepared. During the purification process the manufacturing yield of cinnamon was about 8–9%. Previous studies have used and shown a beneficial effects of 1 g, 3 g and 6 g of cinnamon [8]. Since we are using refined cinnamon (as opposed to crude in other studies), the corresponding doses of refined cinnamon for 1 g, 3 g, 6 g of raw cinnamon was 85 mg (1st month), 250 mg (2nd month) and 500 mg (3rd month), based on the purification yield. The capsule preparation was carried out at a manufacturing site with GMP (Good Manufacturing Practices) certification. The subjects were prescribed CZ capsules for a period of 3 months. Three doses of refined CZ were used (85 mg, 250 mg and 500 mg). Each participant was given 85 mg daily for 30 days during the 1st month (each capsule 85 mg), 250 mg daily for 30 days during the 2nd month (each capsule 250 mg) and 500 mg daily for 30 days during the 3rd month (2 capsules of 250 mg per day).

### Data collection, Pharmacodynamic and safety outcomes

Data collection was carried out by trained medical research assistants, using a standardized case record form, at baseline (Visit ‘0) and during each monthly follow up visit (Visit ‘1’, ‘2′ and ‘3′). Initially a brief history was taken from each patient, which included symptoms related to any disease, subsequently a physical examination was carried out, which included the measurement of body temperature, examination of the systems (respiratory, cardiovascular, abdomen and nervous system). Cardiovascular examination included the measurement of blood pressure (SBP and DBP) and pulse (rate, rhythm and volume). Subsequently anthropometric measurements were carried out, which included the measurement of the height, weight, waist and hip circumferences. The following biochemical assessments were done at baseline, at the stated intervals and on completion of the study (Table 1); FBG, total cholesterol [TC], triglycerides, LDL cholesterol, HDL cholesterol, liver function tests (AST, ALT, PT/INR and serum bilirubin), renal function tests (serum creatinine), urine analysis (UFR) and FBC (white cell count, haemoglobin and platelet count). Furthermore, any adverse events which occurred during the preceding month were also noted during these visits. Drug compliance of patients was evaluated by pill counting (number of pill’s returned monthly). Subjects are asked to return remaining drugs and their compliance will be evaluated by using the formula given below,

$$ \mathrm{Compliance}\ \left(\%\right)=\left[\left(\mathrm{distributed}\  \mathrm{drugs}\hbox{-} \mathrm{remaining}\  \mathrm{drugs}\right)/\mathrm{distributed}\  \mathrm{drugs}\right]\times 100 $$

**Table 1:** Summarized study schedule at each visit in the clinical trial

	Visit 0 (screening visit)	Visit 1 (1 month)	Visit 2 (2 months)	Visit 3 (3 month)
Medical history taking	•	•	•	•
FBG	•	•	•	•
Lipid profile^a^	•			•
Liver function^b^	•	•	•	•
Renal function^c^	•	•	•	•
FBC^d^	•			•
Blood pressure	•	•	•	•
Physical examination^e^	•	•	•	•
Urinanalysis^f^	•			•

^a^total cholesterol, triglyceride, HDL cholesterol; ^b^AST, ALT, total bilirubin, PT/INR; ^c^creatinine; ^d^WBC, RBC, hemoglobin (HGB), hematocrit (HCT), platelet count (PLT); ^e^body weight (kg), height (cm), waist circumference (cm), hip circumference (cm), waist:hip ratio; ^f^Urine microscopy, urine for sugar and protein

The details of items which will be measured at every visit are described in Table 1.

### Anthropometric and blood pressure measurements

Body weight was measured using a calibrated electronic floor scale (SECA 815 by SECA GmbH & Co. Kg. Hamburg, Germany) to the nearest 0.1 kg. Height was measured to the nearest 0.1 cm using an upright plastic portable Stadiometer (SECA 217 by SECA GmbH & Co. Kg. Hamburg, Germany). BMI was calculated as weight (in kilograms) divided by the square of height (in meters). Waist circumference (WC) was measured with a non-elastic tape (SECA 203 by SECA GmbH & Co. Kg. Hamburg, Germany) at a point midway between the lower border of the rib cage and the iliac crest at the end of normal expiration. Similarly, the hip circumference was also measured at widest part of the buttocks in the inter-trochantric level to the nearest 0.1 cm. All anthropometric measurements were made by using standard equipment and following WHO guidelines. Seated blood pressure (SBP and DBP) was measured after a 10-min rest with Omron IA2 digital blood pressure monitors (Omron Healthcare, Singapore).

### Biochemical and statistical analysis

Biochemical tests were performed in the laboratory of the Department of Pharmacology, Faculty of Medicine, University of Colombo. Glucose assay was performed by enzymatic colorimetric (glucose oxidase) method in RxDaytona™ chemical analyzer (Randox Laboratories LTD, Antrim, UK). Total, LDL cholesterol, triglycerides, AST and ALT were analyzed by enzymatic colorimetric method in Mindray BA-88A semi auto-analyzer (Mindray medical International LTD, China). HDL- cholesterol was determined by precipitation method and bilirubin was quantified by spectrophotometric method by Mindray BA-88A semi auto-analyzer (Mindray medical International LTD, China). Serum creatinine was measured by kinetic method, whereas PT/INR and FBC was done by the manual method. Parametric and non parametric statistical tests will be applied using the SPSS version 14 (SPSS Inc., Chicago, IL, USA) and Stata/SE 10.0 (Stata Corporation, College Station, TX, USA) for the data analysis.

## Results

### Sample characteristics

**Fig. 1:**
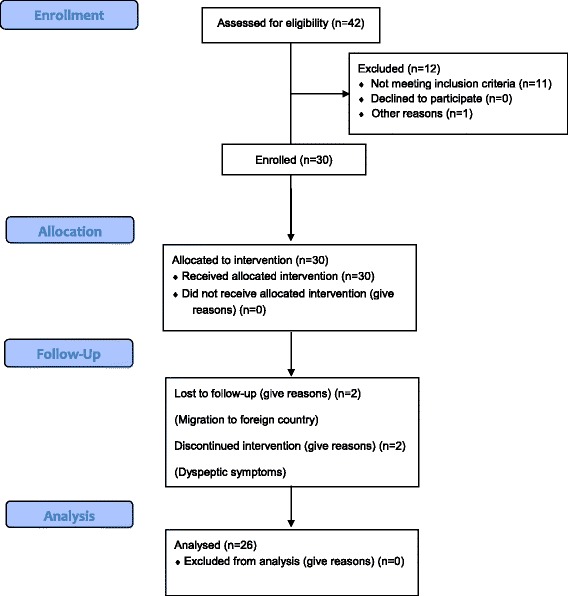
CONSORT Flow Diagram

The total number of subjects recruited for the study was 30, out of which 28 completed the three months follow up (Fig. 1). The mean age (±SD) of the subjects was 38.8 ± 10.4 years (range 21–58 years), and there was a 1:1 gender distribution. Mean (±SD) BMI, waist circumference, hip circumference were 24.8 ± 3.6 kgm^−2^, 86.6 ± 10.6 cm and 97.8 ± 9.0 cm respectively. The mean (±SD) total cholesterol, LDL cholesterol, HDL cholesterol, VLDL cholesterol and triglyceride were 226.4 ± 38.7 mg/dl, 152.8 ± 37.1 mg/dl, 52.7 ± 14.2 mg/dl, 24.6 ± 15.2 mg/dl and 112.1 ± 49.9 mg/dl respectively, while the mean fasting blood glucose level was 91.2 ± 6.9 mg/dl (range 80–104 mg/dl). The mean systolic blood pressure, diastolic blood pressure, pulse rate, serum creatinine, AST, ALT, serum bilirubin and PT/INR were normal level at the baseline. Study population characteristics are summarized in Table 2.

**Table 2:** Baseline characteristics of the study participants

	Mean ± SD	Range
Age (years)	38.8 ± 10.5	21–58
Pulse rate (min^−1^)	73.4 ± 8.4	59–96
Systolic Blood Pressure (mmHg)	124.0 ± 10.8	101–139
Diastolic Blood Pressure (mmHg)	76.8 ± 8.4	55–89
Height (cm)	163.5 ± 6.4	152–178
Weight (kg)	66.5 ± 11.5	41.8–89.2
Body Mass Index (kgm^−2^)	24.8 ± 3.6	18.1–33.8
Waist circumference (cm)	86.6 ± 10.6	64–111
Hip circumference (cm)	97.8 ± 9.0	79–119
Waist to Hip ratio	0.9 ± 0.1	0.7–1.2
Haemoglobin (g/dl)	14.3 ± 1.6	12.2–16.2
White blood cell count (×10^9^ per mm^3^)	6.1 ± 0.5	4.8–6.9
Platelet count (×10^9^ per mm^3^)	202 ± 10.4	187–220
Fasting blood glucose (mg/dl)	91.2 ± 6.9	80–104
Serum creatinine (mg/dl)	0.9 ± 0.2	0.1–1.2
AST (U/l)	27.9 ± 7.3	13–40
ALT (U/l)	21.0 ± 10.9	5–39
Serum bilirubin (mg/dl)	0.8 ± 0.4	0.1–1.7
PT/INR	1.1 ± 0.1	0.8–1.2
Total cholesterol (mg/dl)	226.4 ± 38.7	120–288
LDL cholesterol (mg/dl)	152.8 ± 37.1	67–227
HDL cholesterol (mg/dl)	52.7 ± 14.2	25–86
VLDL cholesterol (mg/dl)	24.6 ± 15.2	12.5–83.4
Triglycerides (mg/dl)	112.1 ± 49.9	42–226

### Clinical and biochemical effects of cinnamon

CZ 85 mg, 250 mg and 500 mg capsules were supplemented over a period of three months, in monthly escalating doses. Clinical and biochemical assessments were done according to the schedule described in Table 1. There were no significant changes in the following anthropometric parameters during the three months follow up; weight, BMI, waist circumference and waist to hip ratio. There was a significant decrease in the hip circumference between baseline (Visit ‘0’) and the first visit (1 month), and also between baseline (Visit ‘0’) and the final visit (3 months) (Table 3). A significant increase in the pulse rate was noted between visits 2 (2 months) and 3(3 months) (*p* < 0.05), however pulse rate remain unchanged between other follow up intervals. Both systolic and diastolic blood pressure reduced significant during the 1st month and this reduction was sustained throughout the 3 months follow up period (Table 3).

**Table 3:** Clinical and biochemical assessment during follow up

	Mean ± SD				P1*	P2*	P3*	P4*
	Visit ‘0’ (Baseline) Screening	Visit ‘1’ (1 month) CZ 85 mg	Visit ‘2’ (2 months) CZ 250 mg	Visit ‘3’ (3 months) CZ 500 mg				
Pulse rate (min^−1^)	73.4 ± 8.4	71.9 ± 9.2	71.5 ± 8.4	75.8 ± 8.6	NS	NS	<0.05	NS
Systolic Blood Pressure (mmHg)	124.0 ± 10.8	118.0 ± 12.8	117.7 ± 13.2	117.3 ± 11.1	<0.001	NS	NS	<0.01
Diastolic Blood Pressure (mmHg)	76.8 ± 8.4	72.7 ± 11.5	71.9 ± 9.1	72.0 ± 8.2	<0.05	NS	NS	<0.01
Weight (kg)	66.5 ± 11.5	66.5 ± 11.2	66.7 ± 11.6	66.4 ± 11.5	NS	NS	NS	NS
Body Mass Index (kgm^−2^)	24.8 ± 3.6	24.8 ± 3.5	24.8 ± 3.7	24.7 ± 3.8	NS	NS	NS	NS
Waist circumference (cm)	86.6 ± 10.6	85.5 ± 9.1	85.6 ± 9.4	85.6 ± 8.8	NS	NS	NS	NS
Hip circumference (cm)	97.8 ± 9.0	96.4 ± 8.4	96.2 ± 8.9	96.0 ± 9.3	<0.05	NS	NS	<0.05
Waist to Hip ratio	0.9 ± 0.1	0.9 ± 0.1	0.9 ± 0.1	0.9 ± 0.1	NS	NS	NS	NS
Haemoglobin (g/dl)	14.3 ± 1.6	NM	NM	14.5 ± 1.8	–	–	–	NS
White blood cell count (*10^9^ per mm^3^)	6.1 ± 0.5	NM	NM	6.3 ± 0.9	–	–	–	NS
Platelet count (*10^9^ per mm^3^)	202.8 ± 10.7	NM	NM	208.4 ± 18.4	–	–	–	NS
Fasting blood glucose (mg/dl)	91.2 ± 6.9	94.5 ± 11.7	93.0 ± 7.6	92.7 ± 9.6	NS	NS	NS	NS
Serum creatinine (mg/dl)	0.9 ± 0.2	0.9 ± 0.2	0.8 ± 0.2	0.9 ± 0.2	NS	NS	NS	NS
AST (U/l)	27.9 ± 7.3	29.7 ± 7.7	29.2 ± 7.5	27.6 ± 6.3	NS	NS	NS	NS
ALT (U/l)	21.0 ± 10.9	23.4 ± 10.9	21.7 ± 10.5	19.2 ± 11.2	NS	NS	NS	NS
Serum bilirubin (mg/dl)	0.8 ± 0.4	0.6 ± 0.3	0.5 ± 0.4	0.7 ± 0.4	NS	NS	NS	NS
PT/INR	1.1 ± 0.1	1.1 ± 0.1	1.1 ± 0.1	1.1 ± 0.1	NS	NS	NS	NS
Total cholesterol (mg/dl)	226.4 ± 38.7	NM	NM	210.24 ± 50.9	–	–	–	<0.05
LDL cholesterol (mg/dl)	152.8 ± 37.1	NM	NM	129.8 ± 47.4	–	–	–	<0.001
HDL cholesterol (mg/dl)	52.7 ± 14.2	NM	NM	61.8 ± 21.4	–	–	–	NS
VLDL cholesterol (mg/dl)	24.6 ± 15.2	NM	NM	27.6 ± 22.7	–	–	–	NS
Triglycerides (mg/dl)	112.1 ± 49.9	NM	NM	115.0 ± 47.9	–	–	–	NS

*ALT* Alanine aminotransferase, *AST* Aspartate aminotransferase, *CZ Cinnamomum zeylanicum, HDL* High Density Lipoprotein, *LDL* Low Density Lipoprotein, *INR* International Normalized Ratio, *NM* Not Measured, *NS* Not Significant, *PT* Prothrombin Time, *VLDL* Very Low Density Lipoprotein; *P1 – Baseline vs Visit 1; *P2 – Visit 1 vs Visit 2; *P3 – Visit 2 vs Visit 3; *P4 – Visit 0 vs Visit 3

In the biochemical assessments, the full blood count (white cells, platelets and haemoglobin), renal function tests (serum creatinine) and liver function tests (AST, ALT, serum bilirubin, and PT/INR), which were normal at baseline, remained within the normal range without any significant alteration during the 3 months follow up. Urine analysis which did not reveal any significant baseline abnormality, remained the same during follow. The mean FBG at the end of the 3 months follow up period was 92.7 ± 9.6 mg/dl, this was not significantly different from the baseline FBG, and there was no difference noted between other follow up intervals (Table 3). When the lipid parameters were considered, the HDL-c, VLDL-c and TG remained unchanged during follow up. However, a significant reduction in the TC (*p* < 0.05) and LDL-c (*p* < 0.001) was noted at the end of the 3 months follow up period.

### Adverse effects, drop outs and compliance

There were no serious adverse effects noted and none of the subjects were hospitalized during the 3 months follow up period. Biochemical assessments evaluating potential target organ toxicity (liver and renal function tests) remained normal throughout the study period. None of the patients experienced any form of hypersensitivity during the study (immediate and/or delayed). Dyspeptic symptoms were noted in 4 study participants, and these symptoms included upper abdominal discomfort, nausea, heartburn and bloating. In two study participant these symptoms were mild and only lasted during the first week of CZ supplementation (85 mg [1st month]) and subsequently disappeared with modification of diet and other life style factors related to exacerbation of dyspepsia.

In the remaining two participants (P_1_ and P_2_) dyspepsia necessitated the discontinuation of study participation. P_1_ was a 51 year old female participant, who experienced dyspeptic symptoms during the 1st month (85 mg), these symptoms gradually worsened and were not altered by modification of diet and other factors, and discontinued participation during the 3rd week of the study. P_2_ was a 49 year old female participant, who experienced dyspeptic symptoms during the 2nd month (250 mg), and did not experience any such symptoms during the 1st month (85 mg). Since the symptoms gradually worsened and were not responsive to lifestyle modifications, participation was discontinued at the end of the 6th week of follow up (1½ months). In all 4 patients who experienced dyspeptic symptoms, only lifestyle modifications were done, and no drugs were co-prescribed.

Drug compliance (%) of patients was evaluated by pill counting, according to the formula described above. The mean % compliance (±SD) during the 1st month, 2nd month and 3rd month were 90.2 ± 11.6 (range 63.3–100), 89.9 ± 11.5 (range 66.7–100) and 88.7 ± 9.7 (range 66.7–100) respectively.

## Discussion

The main objective of the study was to evaluate the medium-long term safety and pharmacodynamic properties of CZ. This study was conducted as a phase I clinical trial over a period of three months, with monthly escalating doses of CZ (CZ 85 mg, 250 mg and 500 mg) in healthy adults. In the present study CZ was given to healthy adults which shown to contain a lesser coumarin content [3, 4], compared to *Cinnamomum cassia*. Several biochemical and clinical parameters were assessed throughout the study to assess pharmacodynamic properties and toxicity. To assess the safety of supplementation of CZ, liver and kidney functioning tests were done at the baseline and at the end of each month. Participants were screened initially and at the end of each month to see any undesirable effects occurred due to the CZ supplement. However there were no such effects other than the dyspeptic symptoms requiring discontinuation of treatment in 2 participants out of 30. A significant reduction in systolic/diastolic blood pressure, TC and LDL-c was noted at the end of the 3 months follow up period.

The fact that CZ reduces arterial blood pressure has been concluded by several in-vivo animal studies. Nyadjeu et al. [9] examined the effects of CZ extracts on mean arterial blood pressure (BP) of normotensive (NR) rats, salt-loaded hypertensive rats (SLHR), L-NAME (Nitro-L-Arginine Methly Ester) induced hypertensive rats (LNHR) (Hypertension was induced by inhibition of NO production using L-NAME) and spontaneously hypertensive rats (SHR). Immediately after intravenous administration a significant drop of BP was shown in NTR, SLHR and LNHR in a dose dependent manner, the drop in BP was not dose dependent in SHR. A study done by Wansi, et al. demonstrated similar effects in NTR and SLHR [10]. The blood pressure reduction of CZ is probably mediated via vasodilatation, which is partially achieved by increasing the endothelial nitric oxide (NO) and by activating the K + -ATP channels (ATP sensitive potassium channels) in vascular smooth muscle [9]. Wansi, et al. showed CZ has a vasodilatation effect on the rat thoracic aortic ring segments, suggesting that, CZ might also be inhibiting extracellular Ca^2+^ through L-type voltage-sensitive channels. Another study evaluating acute and chronic antihypertensive effects of CZ showed methanol extract of CZ was able to increase NO production in cardiovascular organs, namely the aorta and the heart [11]. This study revealed that acute antihypertensive effect of CZ is partially due to acute inhibition of L-NAME (an inhibitor of NO synthesis) and chronic antihypertensive effect is probably to the ability of the active phytomolecules of CZ to inhibit cardiovascular remodeling, to improve arterial wall compliance and to prevent endothelial impairment through its antidyslipidemic effects [11]. Maintenance of a normal blood pressure is dependent on the balance between the cardiac output and peripheral vascular resistance. Most patients with essential hypertension have a normal cardiac output but a raised peripheral resistance [12]. Therefore we can postulate that CZ will reduce arterial blood pressure in hypertensive patients by reducing peripheral vascular resistance as a result of its vasodilatation properties and by the improvement in arterial wall compliance.

In the present study we also observed a significant reduction in the TC and LDL-c at the end of the 3 months follow up period whereas there was no significant change in HDL-c, VLDL-c and TG values throughout the study. However Javed, et al. [13] and Kassaee, et al. [14] have demonstrated that CZ significantly reduces TC, LDL-c and TG while it significantly increases HDL-c in hperlipidemic rabbits and hyperlipidemic hamsters respectively. Similar results were observed in another two studies done in Streptozotocin induced diabetic rats [15, 16]. However, it is important to note that in both these studies Streptozotocin induced diabetic rats had a higher TC, TG, LDL-c and lower HDL-c compared to healthy rats prior to the commencement of CZ supplementation. Another Sri Lankan study has demonstrated that CZ significantly reduces TC and LDL-c whereas no significant change in TG and HDL-c in healthy rats which is similar to our findings [6]. The possible reason for the different findings in these studies could be CZ has the potential to reduce TG levels and improve HDL-c only in dyslipedemic participants. An in-vivo animal study by Javed et al. [13] concluded that CZ methanol extract and Simvastatin are equi-efficacious in the treatment of hyperlipidaemia. However, these results were contradicted by a study among patient with type-2 diabetes which demonstrated CZ supplementation (3 g/day) had no effect on lipid profile [17]. In another study involving patients with type-2 diabetes, supplemented CZ (3 g) for a period of 8 weeks, a significant beneficial effect on total cholesterol, LDL, and HDL level was observed [18]. The mechanism involved in cholesterol lowering activity of CZ may be the inhibition of lipid absorption [19]. Further, increases in the expression of the LDL-R gene following cinnamon consumption might be another mechanism that can explain the lipid-lowering activity [14]. However, since studies have contradictory findings this effect needs to be explored further via clinical trials in humans before drawing conclusions with regards to the anti-hyperlipidaemic activity of CZ.

Present study revealed no significant reduction in FBG as a result of CZ supplementation. In a previous Sri Lankan pre-clinical study, a significant difference in FBG and 2-h post-prandial blood glucose of CZ supplementation was shown in diabetes-induced rats. However, in the same study no change was observed among the healthy rats. A systematic-review and meta-analysis by same study group reported that CZ has the potential to reduce FBG in diabetes (animal study data) and explored the probable mechanisms of blood glucose reduction [20]. However, it is possible that CZ does not cause a reduction in fasting blood glucose among healthy normoglycaemic subjects, as in the present study. A recently concluded human cross-over clinical trial involving healthy volunteers given 1 g of CZ, a significant reduction in the post-prandial glycaemic response was observed after a standard meal [21]. This was observed in the absence of a simultaneous stimulation of insulin secretion [21]. The same study demonstrated that in-vitro CZ inhibited pancreatic alpha-amylase activity, while also acutely reducing the glycaemic response to starch in a dose-dependent manner in rats [21]. These findings have been contradicted by another similar study using CZ (3 g), in healthy volunteers [22]. In the present study the post-prandial response to a glucose load was not evaluated, either by providing a standard meal with the measurement of post-prandial blood glucose, nor a 75-g oral glucose tolerance test. In a cross-over clinical trial involving adults with Impaired Glucose Tolerance (IGT), 6 g of CZ was administered and glycaemic response was measured after a 75-g oral glucose tolerance test [23]. The study did not demonstrate any beneficial effects of CZ, and the authors concluded that the ingestion of CZ does not affect postprandial plasma glucose or insulin levels in human subjects [23]. It is important to note that both studies evaluated the glycaemic response only after a single dose of CZ. In view of promising results observed in in-vitro and in-vivo animal studies and the conflicting results from human trials, further well-designed randomized placebo-controlled medium-long term studies are required prior to drawing conclusions regarding the effectiveness of CZ on glycaemic control.

We did not observe any significant reductions in most anthropometric parameters (weight, BMI, waist circumference and waist to hip ratio) during and after the three months of CZ supplementation. Similar to this, previous studies have also reported no significant change in body weight following CZ supplementation in healthy rats [15, 24].

Our results did not reveal any significant change in full blood count, liver enzymes (AST, ALT) and in serum creatinine levels. These results are in agreement with those obtained by Anand, et al. [25], where no significant increase in the above were noted even at higher doses confirming its higher margin of safety. In addition in-vivo animal studies have revealed that in diabetic rats that had increased levels of AST, ALT and ALP, they were significantly decreased to near normal following CZ treatment [15, 16, 26]. In addition histological analysis of the liver, kidney, and pancreas after CZ supplementation in rats demonstrated that those organs were within normal histological limits with no evidence of toxicity [6]. Current study also evaluated for any possible anticoagulant effect of CZ by assessing PT/INR at different intervals and revealed no changes. This is possibly because of the very low coumarin levels (naturally occurring strong anticoagulant) in CZ compared to Cassia cinnamon. [27]. There were no other serious adverse effects in the participants during the study period. Dyspeptic symptoms were observed in 4 participants, which disappeared with modification of diet in 2 study participants. However, neither dyspepsia nor other side effects reported due to CZ supplementation in two previous studies done in humans and there were no dropouts due to side effects [17, 28]. Furthermore, many studies have shown beneficial effects of CZ against dyspepsia [29–31]. An in-vitro study has also shown that CZ significantly inhibit *Helicobacter pylori* growth [29].

This is the first phase I clinical trial in health adults evaluating efficacy and safety of CZ. Other strengths of this study are supplementation of 3 different doses of CZ, minimum dropout rate and the assessment coagulation, which has not been explored to date. The limitations of the study are the sample size, duration of the study, lack of a control group and not quantifying the active compounds in *Cinnamomum zeylanicum*. Our sample size was 30 as it was difficult to recruit healthy people for a clinical trial since they are reluctant to take a medicinal supplement daily in the absence of any disease conditions. Larger samples may be able to elucidate other side effects and better describe the pharmacodynamic properties of CZ. The study duration was also limited to 3 months, longer durations of supplementation may be required to identify side effects with long term therapy and also to understand the sustenance of the beneficial pharmacodynamic properties observed. This being a Phase I clinical trial, the lack of a control group also needs to be taken in to account, prior to drawing conclusions from its results. Furthermore, we were not able to assess and quantify the active compounds in the CZ extract used in this study, responsible for the pharmacological effects elicited. Identification of active compounds requires further in-vitro and in-vivo evaluation.

## Conclusions

This is the first phase I clinical trial in health adults evaluating efficacy and safety of CZ. Our results demonstrate no significant side effects and toxicity of CZ, including hepatotoxicity and anti-coagulation properties. CZ demonstrated beneficial anti-hyperlipidaemic and blood pressure lowering effects among healthy adults. Further studies with larger samples and longer durations may be able to elucidate other side effects and better describe the pharmacodynamic properties of CZ.

## Abbreviations

ALP: Alkaline Phosphatase
ALT: Alanine aminotranferase
AST: Aspartate aminotranferase
BMI: Body Mass Index
BP: Blood Pressure
DBP: Diastolic Blood Pressure
ECG: Electrocardiogram
FBC: Full Blood Count
FBG: Fasting Blood Glucose
GCP: Good Clinical Practice
GMP: Good Manufacturing Practices
HDL-c: High Density Lipoprotein cholesterol
LDL-c: Low Density Lipoprotein cholesterol
L-NAME: Nitro-L-Arginine Methly Ester
LNHR: L-NAME induced Hypertensive Rats
NM: Not Measured
NO: Nitric Oxide
NR: Normotensive Rats
NS: Not Significant
PT/INR: Prothrombin Time/International Normalized Ratio
SBP: Systolic Blood Pressure
SD: Standard Deviation
SHR: Spontaneously Hypertensive Rats
SLCTR: Sri Lanka Clinical Trials Registry
SLHR: Salt-Loaded Hypertensive Rats
SPSS: statistical package for the social sciences
TC: Total Cholesterol
TG: Triglycerides
UFR: Urine Full Report
WC: Waist Circumference
WHO: World Health Organization
